# Resistance Exercise Training and Greek Yogurt Consumption Modulate Markers of Systemic Inflammation in Healthy Young Males—A Secondary Analysis of a Randomized Controlled Trial

**DOI:** 10.3390/nu17172816

**Published:** 2025-08-29

**Authors:** Emily C. Fraschetti, Ali A. Abdul-Sater, Christopher G. R. Perry, Andrea R. Josse

**Affiliations:** School of Kinesiology and Health Science, Muscle Health Research Centre, Faculty of Health, York University, Toronto, ON M3J 1P3, Canada; ecfrasch@yorku.ca (E.C.F.); aasater@yorku.ca (A.A.A.-S.); cperry@yorku.ca (C.G.R.P.)

**Keywords:** dairy, Greek yogurt, inflammation, resistance exercise, exercise training

## Abstract

Background/Objectives: Chronic exercise training reduces markers of systemic inflammation; however, less is known about how to optimize this adaptation using nutrition. Dairy products, especially fermented ones, like Greek yogurt (GY), contain anti-inflammatory constituents. This secondary analysis aimed to examine the influence of post-exercise GY consumption vs. an isoenergetic carbohydrate pudding (CP; control) on markers of systemic inflammation during an exercise training intervention. Methods: Thirty healthy young males completed 12 weeks of resistance and plyometric exercise training and were randomized to consume GY (*n* = 15) or CP (*n* = 15). Rested/fasted blood samples were acquired at baseline, and weeks 1 and 12, and inflammatory biomarkers (tumor necrosis factor-alpha [TNF-α], interleukin [IL]-6, IL-1 receptor antagonist [IL-1ra], IL-1Beta [IL-1β], IL-10, and C-reactive protein [CRP]) were measured. Linear mixed models were run on the absolute concentrations, and linear regressions were performed on the absolute change (baseline to week 12), allowing us to account for important covariates. Results: In both groups, CRP (pro) and IL-1ra (anti) increased at week 1 vs. baseline and week 12, while IL-1β (pro) decreased at week 12 vs. baseline (main time effects). We observed significant interactions for IL-6, TNF-α, and the TNF-α/IL-10 ratio, indicating that at week 12, IL-6 (pro) was lower in GY, whereas TNF-α and TNF-α/IL-10 (both pro-inflammatory) were higher in CP vs. week 1 and baseline, respectively. Additionally, within our linear regression models, higher baseline concentrations of IL-1ra (anti), IL-10 (anti) and CRP (pro) predicted greater change over the intervention. Conclusions: These results indicate that our intervention benefited circulating inflammatory markers, and GY supplementation may enhance these effects.

## 1. Introduction

Post-exercise nutrition is often a critical component of exercise training and can aid in improving recovery and maximizing training adaptations [1,2,3]. It is well known that chronic exercise training has anti-inflammatory effects and reduces markers of systemic inflammation, including cytokines like interleukin (IL)-6 and tumor necrosis factor-alpha (TNF-α) [4,5]. Incorporation of different types of post-exercise nutrition may further bolster or hinder this positive effect.

Dairy products contain carbohydrates (glycogen resynthesis), high-quality protein (muscle protein synthesis), and electrolytes (rehydration), making them an excellent post-exercise supplement [1,3,6]. Further, dairy products have anti-oxidative [7] and anti-inflammatory constituents [8], and their intake has been shown to reduce (or have a neutral effect on) markers of inflammation (ex., C-reactive protein [CRP] or TNF-α) [9,10,11,12]. While more research has focused on the use of milk as a post-exercise supplement, yogurt may be of interest as Canadians tend to consume more yogurt, and less milk, according to the 2015 Canadian Community Health Survey [13,14]. Yogurt, and particularly Greek yogurt (GY), may also have additional health benefits, due to its fermented nature, additional bioactive components, such as lactic acid bacteria, and higher protein content (*vs*. non-Greek-style yogurt and milk) [15,16]. This may improve immune function (directly and indirectly) by modulating the gut microbiome, gut permeability, and metabolic endotoxemia markers, as well as reducing nuclear factor kappa B (NF-kB) activity, all of which may result in lower circulating inflammatory cytokines [17].

Many studies have found benefits of consuming dairy (i.e., milk or yogurt) during exercise training, including improving strength [18,19,20,21,22,23,24,25] and body composition [18,19,20,25,26] in healthy young males and females compared to carbohydrate controls. However, fewer studies have investigated the influence of dairy products on reducing inflammation during longer-term exercise training in healthy young adults [21,27,28,29]. While one study found no change in white blood cell counts pre- to post-training (2 weeks of jumping rope and outdoor walking daily), regardless of the post-exercise nutrition [28], another study found an increase in total white blood cell counts, attributed to increases in lymphocyte and monocyte counts, after 9 weeks of exercise training (unspecified) in groups consuming whey-enriched probiotic yogurt (vs. non-probiotic, non-whey-enriched yogurt) [21]. Importantly, neither study reported how long after the last training session the samples were obtained; thus, it is unclear whether these samples were taken in a resting state or whether they reflect a phase of the acute post-exercise inflammatory response [21,28]. In terms of shorter-term trials, McKinlay et al. [27] found an increase in IL-10 in the group consuming GY (compared to a carbohydrate pudding), following 5 days of a soccer training camp in adolescents. This may be a possible benefit of GY consumption, as it increased this anti-inflammatory marker; however, these samples were obtained only 12 h following the last exercise session and may still represent the acute inflammatory response to exercise [27]. No effects or interactions were seen for TNF-α or IL-6, but both groups observed an increase in CRP over the 5 days of training [27]. Increases in CRP were also reported during a 15-week running intervention (supervised runs 2 days/week), comparing post-exercise milk consumption vs. post-exercise kefir consumption in healthy, endurance-trained adults [29]. CRP increased only in the milk group, with no change in the kefir group [29]. These results may indicate a particular benefit of fermented dairy products on inflammation, as they were able to mitigate the increase in CRP during rigorous exercise training. Further research investigating the influence of fermented dairy products, specifically Greek yogurt, on markers of inflammation during exercise training is warranted.

While studies have investigated post-exercise dairy consumption following an acute bout of exercise on markers of inflammation [30,31,32,33,34,35,36], less research has examined the use of wholefood dairy consumption, especially GY, during exercise training on inflammatory markers [21,27,28,29]. Furthermore, no studies have investigated both the short-term (cumulative acute response) and long-term (training response) effects of GY consumption and exercise training. Thus, the objective of this investigation was to examine the short-term (1 week) and long-term (12 week) influence of progressive resistance exercise training and GY consumption, compared to consumption of an isoenergetic carbohydrate pudding (CP), on markers of systemic inflammation (IL-6, IL-10, IL-1 receptor antagonist [IL-1ra], IL-1 beta [IL-1β], TNF-α, and CRP) in the rested, fasted state. A secondary objective was to explore how changes in body composition following exercise training impacted the change in inflammatory markers using multiple linear regression. We hypothesized that due to the anti-inflammatory effects of fermented dairy products, exercise training with GY consumption would reduce pro-inflammatory and enhance anti-inflammatory cytokine concentrations more than exercise training and CP consumption.

## 2. Materials and Methods

The present study is a secondary analysis of a parallel randomized controlled trial (clinical trial registration: # NCT03196856, 1 July 2017). Data collection took place from 2017 to 2018. The primary analysis investigated the effect of GY consumption vs. an isoenergetic carbohydrate-based pudding control (CP) with 12 weeks of resistance training on muscle strength and body composition in young healthy males [20]. We also published another secondary analysis from this study, highlighting changes in bone metabolism with GY and exercise [37]. In the present investigation, we examined the influence of the intervention on resting systemic inflammatory markers, including IL-1ra, IL-1β, IL-6, IL-10, TNF-α, and CRP.

### 2.1. Participants

Thirty young (18–25 years) healthy males were recruited from Brock University (St. Catharines, ON, Canada). Participants were naïve to resistance exercise training (0–2 times/week in the last six months), did not take dietary supplements in the last six months, and had a normal body fat percentage (<25%). All participants provided written informed consent. The study was approved by the Brock University Biosciences Research Ethics Board (REB#16-295, 4 July 2017) and conformed to all standards of Canada’s Interagency Panel on Research Ethics for conducting human research.

### 2.2. Supplement Protocol

Upon enrollment, participants were randomized to either GY or CP using an online-generated randomized scheme (https://numbergenerator.org/ [accessed on 7 July 2017]). Study personnel enrolled participants and assigned them to the intervention groups based on the randomization scheme. Participants in GY consumed 200 g of 0% fat Oikos plain GY (~110 kcal, 20 g protein, 8 g carbohydrate, 208 mg calcium, 283 mg phosphorus, 282 mg potassium; Danone Canada Inc., Boucherville, QC, Canada) 3x/day on training days (immediately post-exercise, 1 h post-exercise, and before bed), and 150 g 2x/day on non-training days (breakfast and before bed). At the same times, CP consumed 47 g of a carbohydrate-based pudding (maltodextrin, chocolate pudding powder, and water), made in-house (110 kcal, 0 g protein, 28 g carbohydrate). While participants and study personnel could not be blinded to their group allocation, the contents of the CP were concealed and deemed the “study-designed supplement”. During the study, both groups were encouraged to maintain their habitual diets, except for the intervention food. Participants were provided with the same dietary advice from study personnel to help them compensate for the added calories consumed from the supplements. Additional details regarding the supplementation protocol have been published elsewhere [20].

### 2.3. Training Protocol

Both GY and CP groups underwent 12 weeks of high-intensity, high-impact resistance and plyometric exercise training, 3x/week. Training sessions occurred at Brock University and were facilitated by certified personal trainers and/or trained senior kinesiology students blinded to group allocation to ensure proper lifting form and mitigate injury. Details of the training protocol have been published elsewhere [20]. In brief, each session was approximately 60 min and consisted of either full-body resistance training (2d/week), including leg press, bench press, and seated row (8–10 exercises, 3–4 sets, 8–12 reps at 70% 1-repetition maximum) or plyometric training (1d/week), including box jumps and frog jumps, for 150–200 jumps/impacts per session.

### 2.4. Body Composition

Body composition (fat mass (FM) and fat-free mass (FFM)) was examined prior to and after the 12-week intervention using air-displacement plethysmography via the Bod Pod (COSMED USA Inc., BODPOD, Chicago, IL, USA). All measurements were completed 48–72 h after the last study exercise session, in the morning, following a minimum 10 h fast. Weight was measured on a calibrated digital scale, which is part of the Bod Pod system. Height was measured to the nearest 0.1 cm using a stadiometer. Additional details regarding body composition measures were previously reported [20]. As reported in the primary paper by Bridge et al. [20], there was a main effect of time (*p* < 0.001) for weight, which increased from pre to post, in both groups. There was an interaction (*p* = 0.046) for FFM, which increased more in GY (2.4 kg, 95% CI: 1.5–3.2 kg) vs. CP (1.3 kg, 95% CI: 0.5–2 kg). There were also main effects of group for FM and body weight (*p* = 0.035 and *p* = 0.022, respectively), such that CP had higher fat mass and body weight vs. GY.

### 2.5. Dietary Analysis

Dietary intake prior to the beginning of the study (baseline/week 0) and during the 12th (i.e., the last) week of training was recorded by participants using 7-day and 3-day food records, respectively [20]. Dietary intake was input and analyzed using a diet analysis program (Food Processor, ESHA, Inc., Salem, OR, USA). Differences between groups for specific macronutrients and micronutrients have been reported previously [20].

### 2.6. Inflammatory Marker Analysis

Venous blood samples were collected from a vein in the antecubital fossa at baseline, week 1, and week 12 of the intervention using a standard venipuncture technique. All samples were taken in the morning (between 0800 and 1000 h) and following an overnight fast (10+ h). Importantly, due to the influence of exercise on acute changes in inflammatory cytokines [34], participants did not perform exercise within the 48 h prior to the blood sampling [20]. To control for the influence of nutrient intake before sample acquisition, participants were asked to consume a similar meal the evening before all samples. Blood was collected into a serum separator vacutainer and allowed to clot (~10 min) before being centrifuged at 1300× *g* for 15 min. Serum was aliquoted into cryotubes for storage at −80 °C until analysis. Serum concentrations of IL-1β, IL-6, IL-10, and TNF-α (intra-assay coefficients of variation [CV] were 8.9%, 6.4%, 8.3%, and 5.5%, respectively) were analyzed by Eve Technologies (Calgary, AB, Canada; https://www.evetechnologies.com [accessed on 5 September 2021]) in duplicate using a microbead multiplex assay kit (Milliplex MAP Human high sensitivity T cell panel HSTCMAG-28SK, Millipore Corp, Burlington, MA, USA). Serum concentrations of CRP (intra-assay CV: 3.5%) and IL-1ra (intra-assay CV: 1.7%) were analyzed using automated ELISAs (Ella, Protein Simple, Bio-Techne, Minneapolis, MN, USA).

### 2.7. Statistical Analysis

Linear mixed models were conducted in R (R Core Team, Vienna, Austria, version 4.5.1) for Mac OS, using the lme4, lmerTest, emmeans, and effectsize packages. Linear regression analyses were conducted using SPSS V29 (SPSS, Chicago, IL, USA). Significance was set to *p* ≤ 0.05 for all tests. However, differences were considered important if *p* ≤ 0.100 and the corresponding effect size was medium or greater. This method of interpretation follows suggestions from statisticians regarding the use and interpretation of *p*-values urging scientists to not base scientific conclusions on *p*-values alone and to use other metrics or tests to support interpretations of *p*-values (e.g., effect sizes, confidence intervals) [38], particularly with smaller sample sizes in human interventions. While this differs from traditional statistical interpretations in exercise science, this is carried out in an effort to move beyond interpreting data using *p*-values as a dichotomous variable and is performed objectively, providing the reader with a variety of information (i.e., *p*-value, effect size, mean difference) to interpret the data themselves [38], as performed previously in [39]. The figures were made using Prism 10.2.3 for Mac OS (GraphPad software, San Diego, CA, USA). Values are presented as mean ± standard error (SE) for Figures, ± standard deviation (SD) for Tables and confidence intervals (CIs) are presented for the linear regression analyses.

#### 2.7.1. Linear Mixed Models on Dietary Data

Differences between baseline characteristics (age, weight, height and BMI) were assessed using independent *t*-tests. Linear mixed models were used to assess total daily nutrient intake (for calories, macronutrients, and select micronutrients) over the intervention with fixed effects of time (baseline and week 12) and group (GY and CP) and group x time interactions, and a random factor of participant ID. Food diaries were missing for 2 participants in the GY group, and missing data were dealt with within the mixed model using restricted maximum likelihood (REML) estimation. Following a significant interaction, post hoc analyses were conducted (paired and independent *t*-tests) and corrected using Tukey’s Honest Significant Difference.

#### 2.7.2. Linear Mixed Models on Absolute Data

**Figure 1:**
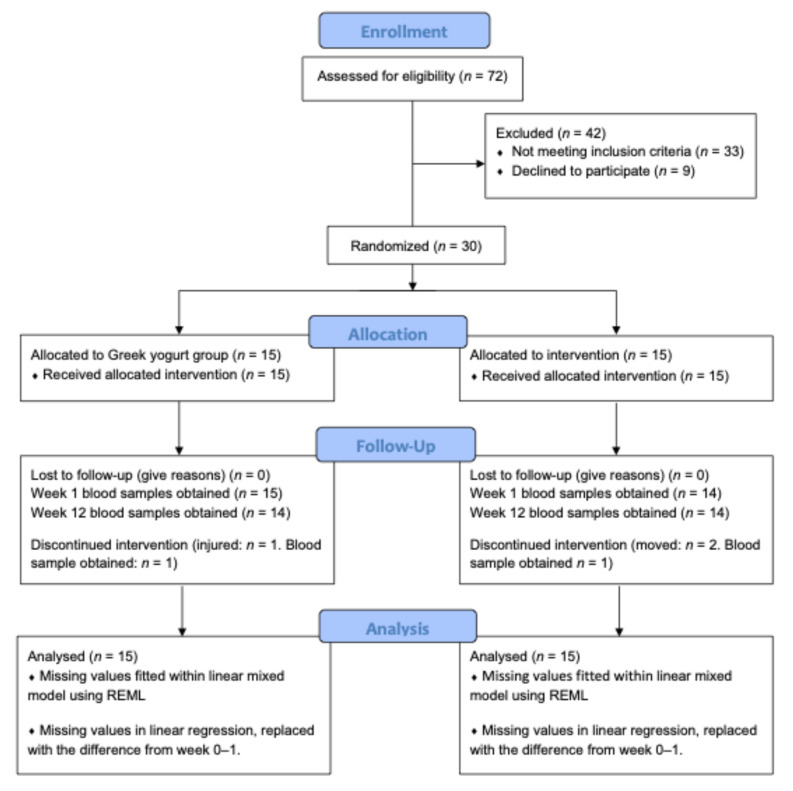
CONSORT diagram depicting the participant flow through the study. REML: restricted maximum likelihood.

Prior to analysis, data were examined for outliers, and values ± >2 SDs (*n* = 29, representing 5.4% of 540 total datapoints) from the mean were replaced with the 2 SD upper/lower limits, respectively. Following outlier treatment, data were assessed for normality using skewness and kurtosis scores (>3). IL-1ra, IL-10, CRP, and the ratio of TNF-α/IL-10 were not normally distributed, so these data were log-transformed prior to statistical analysis. We conducted linear mixed models on the absolute concentrations of the inflammatory markers with fixed factors of time (baseline, week 1, and week 12) and group (GY and CP) and group x time interactions, and a random factor for participant ID. As we were unable to obtain blood samples for one participant at week 1 (CP) and two participants at week 12 (1 from GY, 1 from CP), missing data within the mixed model were dealt with using REML estimation (Figure 1). Following a significant *F*-test, post hoc analyses were conducted (paired and independent *t*-tests) and corrected using Tukey’s Honest Significant Difference. Partial eta-squared (η_p_^2^) values were calculated to estimate the effect sizes (small: 0.01, medium: 0.06, and large: 0.14) for main effects and interactions. Cohen’s *d* (*d*) values were calculated to estimate the effect sizes (small 0.2, medium 0.5, large 0.8, very large 1.3) for post hoc comparisons [40].

#### 2.7.3. Linear Regression

To explore what variables (and to what extent) may have influenced the change (baseline to week 12) in each cytokine over the entire intervention, linear regression models were evaluated. In each group, there were missing blood samples for one participant at week 12; in these cases, the change from baseline to week 1 was used. The variables entered into the models included group (GY and CP), the absolute change (from baseline to week 12) in FM and FFM (due to the influence of body composition on inflammation [41,42]), the absolute change in each inflammatory marker, and the baseline concentration for the respective marker. Absolute change (rather than % change) was utilized to be consistent across all markers. Using backwards regression, models with the highest adjusted *R*^2^ were selected and presented herein.

## 3. Results

### 3.1. Baseline Characteristics

Baseline characteristics for both groups are presented in Table 1. There were no differences between groups for age, height, weight, or BMI at the start of the intervention.

### 3.2. Average Daily Dietary Intakes (Table 2)

Comparisons of total energy intake, macronutrients, and select micronutrients (particularly dairy-containing micronutrients) are presented in Table 2. Across the intervention, both groups increased their daily energy, magnesium, and potassium intake (main effects of time, *p* < 0.05). At week 12, GY was consuming more protein, calcium, and phosphorus compared to baseline and compared to CP (post hoc tests *p* < 0.001, for all). At week 12, CP was consuming more carbohydrates vs. baseline (*p* < 0.001), but was not different vs. GY (*p* = 0.213).

### 3.3. Absolute Cytokine Concentration Analysis (Figure 2)

#### 3.3.1. Anti-Inflammatory Cytokines

**Figure 2:**
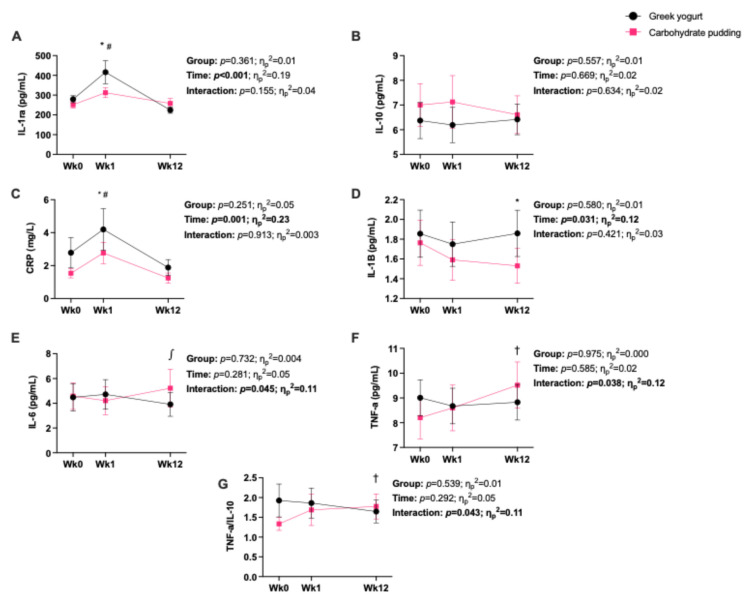
The absolute concentrations of interleukin-1 receptor antagonist (IL-1ra) (**A**), IL-1 beta (IL-1β) (**B**), IL-6 (**C**), IL-10 (**D**), C-reactive protein (CRP) (**E**), tumor necrosis factor-alpha (TNF-α) (**F**), and the ratio of TNF-α/IL-10 (**G**) over the 12-week (Wk) intervention (baseline/Wk0, Wk1, Wk12) for both Greek yogurt (GY) and carbohydrate pudding groups (CP). Data are presented as mean ± standard error. Symbols denote a significant difference from baseline (*) or week 12 (#) for both groups, a difference from baseline in CP only (†), and a difference from week 1 in GY only (*∫*). *p*-values and partial eta-squared effect sizes for the main effects and interactions are displayed.

The concentration of IL-1ra increased at week 1 vs. baseline and week 12 (main effect of time; post hoc tests, *p* = 0.012, *d* = 0.776 and *p* < 0.001, *d* = 1.121, respectively; Figure 2A). There were no main effects or interactions for IL-10 (Figure 2B).

#### 3.3.2. Pro-Inflammatory Cytokines

The concentration of CRP increased at week 1 vs. baseline and week 12 (main effect of time; *p* = 0.037, *d* = 0.664, *p* = 0.001, *d* = 1.121, respectively; Figure 2C). The concentration of IL-1β decreased at week 1 (*p* = 0.107, *d* = 0.540) and week 12 (*p* = 0.036, *d* = 0.674), compared to baseline (main effect of time; Figure 2D). We observed an interaction for IL-6 (Figure 2E), whereby the concentration of IL-6 decreased at week 12 vs. baseline (*p* = 0.108, *d* = 0.772) and week 1 (*p* = 0.027, *d* = 1.00) in GY and did not change in CP. We observed significant interactions for the absolute concentration of TNF-α (Figure 2F) and the TNF-α/IL-10 ratio (Figure 2G), whereby at week 12 the concentration and ratio increased in CP (post hoc tests, *p* = 0.038, *d* = 0.947 and *p* = 0.014, *d* = 1.096, respectively) but not in GY.

### 3.4. Linear Regression for the Change in Each Inflammatory Marker (Table 3)

A higher baseline IL-1ra predicted a greater decrease in IL-1ra over the intervention. Similarly, a higher baseline IL-1β predicted a greater decrease in IL-1β (*p* = 0.053; CI: −0.297–0.002). Also, for IL-1β, a greater increase in FM over the 12 weeks predicted a decrease in IL-1β. For IL-6, compared to CP, GY predicted a decrease in IL-6, and an increase in FFM predicted an increase in IL-6, over the 12-week intervention. The change in TNF-α and the baseline concentration of IL-10 predicted increases and decreases, respectively, in IL-10 over the intervention. Furthermore, there appeared to be an influence of group (*p* = 0.081; CI: −0.097 to 1.528), where GY predicted an increase in IL-10, compared to CP. For CRP, a higher baseline concentration of CRP predicted a greater decrease in CRP over the intervention. For TNF-α, GY predicted a decrease in TNF-α, compared to CP, and an increase in IL-10 predicted an increase in TNF-α.

## 4. Discussion

Consumption of GY during 12 weeks of resistance exercise training improved some resting inflammatory marker concentrations compared to CP in healthy young males. GY resulted in a reduction in IL-6 (from week 1) and mitigated the increase in TNF-α and the TNF-α/IL-10 ratio seen in CP. These results were confirmed in our linear regression analysis, whereby GY consumption led to greater reductions in IL-6 and TNF-α over the 12-week intervention in GY vs. CP, even after controlling for body composition and the change in other inflammatory markers. Thus, consumption of GY during an exercise training intervention may provide additional benefits, beyond exercise training with CP consumption, in improving resting systemic inflammation in healthy young males who do not have elevated levels of systemic inflammation. We also observed that, following the initial week of training (i.e., after the first three acute exercise bouts), CRP concentrations increased, indicating an increased inflammatory response to exercise in the short term, but this was restored to baseline values by the end of the intervention. In contrast, within the first week, IL-1ra concentration increased (i.e., indicating a more anti-inflammatory response to exercise in the short term), and IL-1β decreased after 12 weeks of training in both groups. Since our participants were initially naïve to resistance exercise, these results indicate benefits of exercise training, with both GY and CP, in modulating markers of inflammation over the whole 12-week intervention (as decreased IL-1β), during the first week of the intervention (as increased IL-1ra), and in the last 11 weeks of the intervention (as a resolution of increased CRP).

Within our study, we observed several interactions with IL-6, TNF-α, and the ratio of TNF-α/IL-10, indicating that inflammation changed differently in GY and CP over the intervention. Resting IL-6 decreased (from week 1 to week 12) in GY only, indicating a beneficial adaptation. In contrast, another study investigating fortified milk (2 × 200 mL milk/day, providing 1000 mg/day calcium and 800 IU/day vitamin D) with and without exercise (progressive resistance training, 3 days/week) as part of a 2 × 2 factorial design in older adults observed a trending main effect of milk consumption, with IL-6 values increasing over the 18-month intervention [43]. However, the increase was not sustained when the results were adjusted for change in FM over the intervention [43]. Similarly, in our study, the GY and CP groups had different changes in body composition, with GY having greater increases in FFM over the intervention [20]. Our IL-6 results indicated an interaction (*p* = 0.045; Figure 2), with a medium effect size for the interaction (η_p_^2^ = 0.11) and a large effect size for the decrease between weeks 1 and 12 in GY (post hoc; *p* = 0.027; *d* = 1.00). Alongside this, we observed an influence of group (greater decreases in GY vs. CP, over the 12 weeks) within the linear regression, which controlled for the change in FFM. Therefore, it is likely that the change in resting IL-6 was due to the nutritional intervention, and not due to changes in body composition, as seen in [43].

While we did not observe any difference between GY and CP for the anti-inflammatory cytokine, IL-10, we did observe a difference in the TNF-α/IL-10 ratio, such that the ratio for CP increased (more inflammatory) at week 12 vs. baseline (large effect, *d* = 1.096). This CP response was driven by the increase in resting TNF-α that was not met with a concomitant increase in anti-inflammatory IL-10. Investigating ratios of inflammatory cytokines improves our understanding of the overall inflammatory environment, as cytokines up- and down-regulate one another in an interconnected system [4]. For example, production of IL-10 has been shown to inhibit TNF-α, among other pro-inflammatory cytokines [4]. Indeed, within our linear regression analyses, TNF-α and IL-10 are positively related. While no other training and dairy supplementation studies have investigated cytokine ratios, some have examined IL-10 independently following post-exercise nutrition [27,44,45,46]. McKinlay et al. observed an acute increase in IL-10 following GY consumption (vs. CP) after 5 days of intense soccer training in adolescent female athletes [27] and 8 h post-exercise following whey protein supplementation (vs. water) in adolescent swimmers [47]. Both studies indicate an increase in the acute/short-term anti-inflammatory environment with dairy consumption. In the present study, when controlling for the change in other cytokines (via linear regression), we observed a trending group effect (*p* = 0.081) such that IL-10 increased more in GY vs. CP over the 12-week intervention.

We observed an increase in resting TNF-α in CP at week 12 vs. baseline (medium effect, *d* = 0.706), which was not seen in GY. Most other training studies investigating the influence of dairy on inflammatory markers have found no change in TNF-α over the intervention and no difference between nutritional interventions [27,43,44,45,48,49]. However, one study investigating the influence of training and fermented milk on inflammation following a marathon found that those consuming fermented milk (vs. regular milk) for 30 days prior to the marathon mitigated the 2-fold increase in TNF-α immediately following the race [46]. This provides supporting evidence for the use of fermented dairy products, such as yogurt, to modulate the inflammatory response to exercise. While many studies have shown that dairy products can modestly reduce markers of inflammation [9,10,11,12], likely owing to their anti-inflammatory [8] and anti-oxidative properties [7], fermented dairy products, such as yogurt, are of particular interest due to their additional bioactives and bacterial cultures. Indeed, a meta-analysis by Bordoni et al. (2017) reported a greater anti-inflammatory effect for fermented dairy products compared to non-fermented dairy products [50]. It is postulated that yogurt consumption, in particular, can improve immune health through indirect and direct action on the gut, specifically through reducing gut permeability, increasing microbiota diversity, and reducing gut endotoxin (lipopolysaccharide) translocation [17]. It is also possible that the increase in TNF-α in CP was due to the provision of carbohydrates from the pudding over the intervention. However, we did not observe increases in other inflammatory markers assessed. Moreover, it is common for individuals to consume easily digestible carbohydrates post-exercise for energy replenishment [51].

We observed an increase in CRP following 1 week of exercise training (three acute exercise bouts), regardless of nutritional intervention. This was similarly demonstrated by McKinlay et al. during 5 days of intense soccer training [27]. This makes sense as acute exercise can cause inflammation [52]. Performance of three acute sessions at the start of the intervention, particularly in untrained individuals who are unaccustomed to resistance exercise, may result in higher inflammation due to the compounded and sustained muscle damage, even if samples are taken in a rested state (i.e., 48 h post-exercise as was carried out herein). By the end of our intervention (12 weeks), CRP concentrations returned to baseline values as the body positively adapted to the exercise program. Similarly, with IL-6, we observed a reduction in concentration at week 12 from week 1, in GY only. In both instances, we did not see further decreases (beyond baseline) over the intervention in CRP or IL-6. Importantly, our participants were young, lean, healthy males who did not have elevated levels of resting systemic inflammation. Thus, this may relate to a ‘flooring effect’ in their systemic cytokines. Exercise training is most beneficial at improving markers of inflammation in individuals with chronically elevated inflammation, such as those with chronic disease or obesity [53], and there is only a small benefit for healthy, normal-weight individuals [53,54]. This was observed within our linear regression models as greater levels of CRP at baseline predicted greater decreases in CRP over the intervention. This was also the case for IL-1ra and IL-10. As such, to better assess the adaptations of the immune system to exercise and nutrition, particularly in those with lower resting inflammation, it may be beneficial to administer an inflammatory challenge before and after the intervention to investigate the effect of the intervention following a physiological stressor.

It has been long established that higher concentrations of inflammatory markers are typically associated with higher BMIs, greater adipose tissue volume, and obesity [41,42,55,56]. Weight and/or fat mass loss has also been shown to reduce levels of systemic inflammation [55,57,58]. Our group has previously demonstrated that weight loss through diet and exercise in young women with overweight/obesity decreased IL-6 and TNF-α [59], and that reductions in adiposity were associated with reductions in TNF-α following a 12-week diet and exercise intervention in adolescents with overweight/obesity [58]. Within the present study, through our linear regression analyses, we observed that an increase in fat mass was associated with a decrease in IL-1β over the 12-week intervention. This result challenges our understanding of the relationship between fat mass and inflammation [41,60]. However, examining context is important here as these young males were already lean, and a 1 kg increase in FM was only associated with a 0.08 pg/mL decrease in resting IL-1β, which may not be clinically relevant. Specifically, over the intervention, the GY group lost an average of 0.5 kg of fat mass, whereas the CP group gained 0.3 kg [20]. Using this relationship, this would yield a 0.04 pg/mL increase and a 0.02 pg/mL decrease in IL-1β, respectively. This has minimal impact when examining the actual change in IL-1β over the intervention period for the overall group, where we observed an average reduction of 0.23 pg/mL from baseline to week 12 (medium effect, *d* = 0.674). We also observed a positive relationship between FFM and IL-6, such that each 1 kg increase in FFM was associated with a 0.42 pg/mL increase in resting IL-6 over the 12 weeks. Typically, higher levels of IL-6 at rest are associated with lower levels of muscle mass [61]; however, this may not hold true in the context of a progressive exercise training program, due to the production and secretion of IL-6 from muscle, which has crucial roles in initiating the acute inflammatory cascade to promote regeneration and repair of muscle [62].

Our study had some limitations. First, the study was not primarily designed to investigate systemic inflammation. As such, we may be underpowered to detect differences between our groups. Thus, we relied on both *p*-values and effect sizes or confidence intervals for interpretation. Further, as our participants were young, lean, healthy individuals that did not have higher resting levels of inflammation, we are unable to comment on the potential of this intervention to reduce most inflammatory cytokines (beyond baseline) as was observed in other populations following exercise interventions [63,64]. Lastly, while we wanted to investigate the influence of different nutritional supplements on resting systemic inflammation during exercise training, we did not include an exercise-only group (i.e., with no specific post-exercise nutrition) or a no-exercise group (i.e., a no-intervention control group). Both may have provided additional insight into the separate intervention effects of exercise training and nutrition on resting circulating inflammation.

## 5. Conclusions

In conclusion, exercise training provided some anti-inflammatory benefit by reducing resting IL-1β and CRP concentrations (from week 1) in both groups, and consumption of GY (vs. CP) with exercise training decreased resting IL-6 from weeks 1 to 12 (although not beyond baseline) and mitigated the increase in resting TNF-α and TNF-α/IL-10 ratio at week 12 seen in CP. These effects were confirmed in our linear regression, as GY had greater reductions in resting IL-6 and TNF-α from baseline to week 12 compared to CP. Through our linear regression models, we also observed that changes in inflammatory markers over the intervention were influenced by other predictors, such as changes in body composition (FM and FFM) and their respective baseline concentrations. Future research should continue to investigate key predictors for the change in inflammatory markers with exercise and nutrition, the effect of fermented dairy on other markers of immune function with exercise, and the response of these markers following a physiological stressor in the context of an exercise intervention.

## Figures and Tables

**Table 1:** Baseline (week 0) characteristics for both groups.

	CP (*n* = 15)	GY (*n* = 15)	*p*-Value
Age (years)	20.3 ± 2.09	20.9 ± 2.26	0.500
Height (m)	1.78 ± 0.05	1.79 ± 0.06	0.782
Weight (kg)	69.7 ± 10.0	68.3 ± 10.9	0.710
BMI (kg/m^2^)	22.0 ± 2.7	21.3 ± 2.8	0.576
FM (kg)	9.35 ± 4.71	12.23 ± 5.82	0.161
FFM (kg)	58.9 ± 8.61	57.5 ± 6.69	0.638

Note: CP: carbohydrate pudding, GY: Greek yogurt, BMI: body mass index, FM: fat mass, FFM: fat-free mass. Similar values have been reported previously in [20,37].

**Table 2:** Average daily nutrient intakes for each group at baseline (week 0) and week 12.

Nutrient Intake per Day	Greek Yogurt (GY)		Carbohydrate Pudding (CP)		Group	Time	Int
	Week 0	Week 12	Week 0	Week 12			
Calories (kcal)	2150 ± 379	2306 ± 484	1989 ± 384	2303 ± 569	0.357	**0.010**	0.429
Protein (g)	93 ± 19	130 ± 22 * ^#^	86 ± 14	86 ± 19	0.279	**0.001**	**0.001**
Carbohydrate (g)	248 ± 49	259 ± 74	227 ± 52	294 ± 71 *	0.374	**0.002**	**0.040**
Fat (g)	79 ± 17	79 ± 19	80 ± 27	84 ± 35	0.939	0.651	0.539
Calcium (mg)	700 ± 248	1132 ± 331 * ^#^	688 ± 213	657 ± 272	0.910	**<0.001**	**<0.001**
Phosphorus (mg)	764 ± 289	1266 ± 323 * ^#^	668 ± 321	708 ± 339	0.429	**<0.001**	**<0.001**
Magnesium (mg)	174 ± 82	204 ± 83	143 ± 75	178 ± 100	0.346	**0.002**	0.789
Iron (mg)	13 ± 6	14 ± 7	12 ± 4	13 ± 7	0.457	0.305	0.999
Sodium (mg)	3436 ± 1121	3238 ± 1023	3384 ± 1111	3481 ± 1712	0.916	0.727	0.461
Potassium (mg)	1565 ± 669	2017 ± 632	1533 ± 640	1627 ± 1151	0.917	**0.018**	0.092
Vitamin D (mcg)	2 ± 1	2 ± 2	2 ± 1	3 ± 2	0.288	0.365	0.176
Vitamin K (mcg)	45 ± 40	41 ± 57	90 ± 195	55 ± 91	0.302	0.515	0.553

Note: Average daily nutrient intakes were calculated from 7-day (baseline/week 0) and 3-day (week 12) food diaries. Intakes at week 12 include the Greek yogurt and carbohydrate pudding supplements. Two participants were missing dietary data for week 12 in the Greek yogurt group, and missing data were dealt with within the linear mixed model using restricted maximum likelihood estimation. *p*-values for group, time, and interaction (int) are displayed, and values < 0.05 are bolded. For significant interactions, symbols denote a difference from baseline within a group (*) and a difference from CP (^#^; i.e., between groups). Nutrient intakes are expressed as mean ± standard deviation. Similar values have been reported previously in [20,37].

**Table 3:** Multiple linear regression models for the absolute change (baseline to week 12 change [∆]) in each inflammatory marker.

Inflammatory Marker	β	95% CI	*p*-Value
∆IL-1ra			
Group	−40.636	−104.816–23.543	0.205
∆FM	−8.352	022.929–6.226	0.250
Baseline IL-1ra	−0.990	−1.453–−0.522	**<0.001**
∆IL-1β			
∆FFM	0.062	−0.015–0.140	0.111
∆FM	−0.076	−0.138–0.013	**0.019**
∆IL-6	−0.050	−0.119–0.018	0.143
∆IL-10	0.078	−0.020–0.176	0.114
Baseline IL-1β	−0.148	−0.297–0.002	**0.053**
∆IL-6			
Group	−1.385	−2.728–−0.43	**0.044**
∆FFM	0.417	0.011–0.824	**0.045**
∆IL-1β	−1.052	−2.642–0.539	0.186
∆IL-10			
Group	0.716	−0.097–1.528	**0.081**
∆FFM	−0.170	−0.393–0.052	0.128
∆CRP	−0.146	−0.331–0.039	0.117
∆TNF-α	0.410	0.172–0.649	**0.002**
Baseline IL-10	−0.215	−0.331–−0.099	**<0.001**
∆CRP			
∆TNF-α	0.210	-0.046–0.467	0.104
Baseline CRP	−0.615	−0.771–0.460	**<0.001**
∆TNF-α			
Group	−1.778	−2.807–0.749	**0.002**
∆FFM	0.220	−0.087–0.526	0.153
∆FM	−0.173	−0.409–0.064	0.146
∆IL-10	0.611	0.221–1.000	**0.003**

Note: The effect of group is relative to the carbohydrate pudding. Changes (∆) in fat-free mass (FFM) and fat mass (FM) are reported in kg, changes (∆) in interleukin (IL)-1 receptor antagonist (IL-1ra), IL-1 beta (IL-1β), IL-6, IL-10, and tumor necrosis factor-alpha (TNF-α) are reported in pg/mL, and changes (∆) in C-reactive protein (CRP) are reported in mg/L. β refers to the unstandardized Beta coefficient. *p*-values < 0.100 are bolded.

## Data Availability

The data sets presented in this article are not readily available. Requests to access the data sets should be directed to A.R.J., ajosse@yorku.ca.

## Abbreviations

The following abbreviations are used in this manuscript:

GY	Greek yogurt
CP	Carbohydrate pudding
TNF-α	Interleukin
IL	Interleukin
IL-1ra	Interleukin 1 receptor antagonist
IL-1β	Interleukin 1 Beta
BMI	Body mass index
FM	Fat mass
FFM	Fat-free mass
REML	Restricted maximum likelihood
